# Multi-unit recordings in the stick insect *Carausius morosus* reveal distinct mechanosensory and olfactory activity patterns among antennal lobe neurons

**DOI:** 10.3389/fphys.2026.1873043

**Published:** 2026-07-22

**Authors:** Andrea Gonsek, Claudia Groh, Volker Dürr, Martin Strube-Bloss

**Affiliations:** 1 Department of Biological Cybernetics, Faculty of Biology, Bielefeld University, Bielefeld, Germany; 2 Behavioral Physiology and Sociobiology (Zoology II), Biocenter, University of Würzburg, Würzburg, Germany

**Keywords:** antennal lobe, insect, mechanosensory, multimodal integration, olfactory processing, multi-unit-recordings

## Abstract

In the absence of light, the stick insect *Carausius morosus* relies on sensory modalities such as olfaction and mechanosensation instead of vision. Within the near-range environment, these insects use their long antennae to detect obstacles and gather tactile information, enabling them to grasp contacted objects with their front legs. However, for far-range orientation, they may integrate mechanosensory inputs with additional cues from non-contact modalities, including olfaction. Here we focus on olfactory processing in stick insects, aiming to establish the stick insect as a model to study both, *near-range* (tactile) and *far-range* (olfactory, wind, sound) sensory integration and their effects on motor control. As a first step, we established long-term multi-unit recordings of neurons in the primary olfactory neuropile, the antennal lobe (AL) to confirm general principles of odour processing in insects. Indeed, our recordings revealed that hexanal, i.e. the smell of freshly cut leaves, induced a distinct pattern of activity within the AL network, corresponding to its relevance to a herbivore and confirming the significance of the AL in extracting behaviourally relevant olfactory information. Furthermore, we discovered mechanically induced activity in AL neurons likely related to airborne vibration such as sound, highlighting that both olfactory and mechanical stimuli are processed within the AL, with a substantial fraction of neurons responsive to both modalities.

## 1 Introduction

In the absence of light, nocturnal insects like the stick insect *Carausius morosus* cannot rely on visual cues for spatial orientation. Instead, they depend on sensory modalities such as olfaction and mechanosensation, including wind and touch. The stick insect utilizes its long antennae to detect and sample obstacles based on tactile cues (Krause and Dürr, 2012). Upon contacting an obstacle, it can move its front leg to directly grasp it (Schütz and Dürr, 2011). This *near-range* touch-induced targeted motor response is facilitated by the integration of tactile and proprioceptive inputs in descending first-order interneurons (Ache and Dürr, 2013; Ache et al., 2015; Lepreux et al., 2019; Jaske et al., 2021). However, for goal-oriented *far-range* movement, motivating factors like locating food or oviposition sites are crucial, particularly for nocturnal insects that use chemosensory cues (Balkenius et al., 2006; Stöckl et al., 2016). They may also integrate chemoreception with mechanosensory cues like wind or distant sounds to create a multimodal environmental representation. For example, in the moth *Manduca sexta*, it was shown that oscillatory wind dynamics, such as those imposed by the wing beat, entrain odour stimuli and increase neural perception (Tripathy et al., 2010). It might also be possible that predator sound modulates their olfactory perception, as described in the moth *Spodoptera littoralis*, were the sound of a predatory bat sensitize olfactory neurons at the AL level (Anton et al., 2011). To establish the stick insect as a paragon for the integration of both *near-range* and *far-range* sensory information and its effects on motor behaviour, the first objective of the present study is to explore early olfactory processing in stick insects. We do so with long-term multi-unit recordings of antennal lobe (AL) projection neurons (PN) during stimulation with various odour components, characterising their neural representation within the stick insects’ AL network. For all odour components tested, we first proved their detectability and, thus, ecological relevance, via electroantennograms (EAG). Since stick insects are herbivores, we included hexanal, which belongs to a group of green-leaf volatiles that is released when leaves are damaged. Moreover, hexanal has been suggested to act as a direct and indirect defence mechanism of plants against herbivores (Arimura et al., 2005). All other tested odours were chosen so as to allow comparisons with evidence from other insect species, including components of other plants, flowers or pheromones.

Insect antennae carry a wide range of receptor types from different sensory modalities, and serve as actively movable, multisensory organs (Dürr et al., 2022). While the stick insect antenna has been studied mainly with regard to near-range tactile and proprioceptive integration in sensorimotor behaviour (Krause and Dürr, 2012; Krause et al., 2013; Berendes and Dürr, 2022; van der Veen et al., 2026), our knowledge about far-range mechanosensory and olfactory processing and their multimodal integration is missing. Our second objective is to fill this gap.

With regard to olfaction, sensory integration in insects is known to occur early in thedeutocerebrum, close to the periphery. Antennal olfactory receptor neurons (ORN) project to the AL, the primary olfactory processing centre, akin to the olfactory bulb (OB) in vertebrates (Wilson and Mainen, 2006; Hildebrand and Shepherd, 1997). The AL comprises glomeruli, formed by axon terminals of ORNs, local interneurons (LN), and PNs, that encode odours through distinct spatio-temporal excitation and inhibition patterns, modulated by various pre- and postsynaptic LN populations (Galizia and Menzel, 2000; Sachse and Galizia, 2002, 2003; Seki et al., 2010; Strube-Bloss et al., 2017; Grabe et al., 2020). Variation of signal strength in LNs is thought to reflect stimulus-specific information for species-specific functions (Martin et al., 2011) and relative PN activity is thought to encode an odour stimulus (Sachse and Galizia, 2003). Behaviourally relevant information, such as attractiveness or repellence, is clearly represented within the AL network (Strube-Bloss et al., 2015; Knaden et al., 2012; Ronderos et al., 2014).

With regard to mechanosensory processing in conjunction with olfaction, there is increasing evidence in mammals and insects that mechanical stimuli are processed within the olfactory pathway, too. In mice, patch-clamp recordings of OSNs in the OB demonstrated dual processing of both modalities within the same neurons actually mediated by a shared second-messenger cascade (Grosmaitre et al., 2007). In rats, calcium imaging revealed mechanically driven activity in a significant fraction of OB glomeruli also involved in odour coding (Carey et al., 2009). Similarly, in honeybees and cockroaches, mechanically induced activity affects glomeruli to a similar extent as odour-induced activity (Tiraboschi et al., 2021; Zeiner and Tichy, 1998; Patel et al., 2022), indicating a close connection between both modalities. Here, we test for evidence of this bimodal connection in stick insects. We do so with a control stimulus that is generated by switching the solenoid valves of the odour delivery system, either with or without connection to the airflow tubing. Whereas the prior controlled for potential non-olfactory responses related to changes in airflow, the latter controlled for potential effects of airborne vibration, as caused by the “click” sound of the valves.

Our results show that olfactory processing in the stick insect AL is very similar to that found in other insects, with a distinct separation of hexanal-induced activity from that induced by other odorants. Concerning bimodal processing, we show that the majority of recorded AL-neurons are sensitive to both olfactory and mechanosensory stimuli.

## 2 Materials and methods

To study olfactory processing in the stick insect *Carausius morosus*, we combined (i) electro-antenno-graphical recordings (ERG) with (ii) extracellular multi-unit recordings of antennal lobe (AL) neurons during stimulation with five ecologically relevant odour stimuli and (iii) an anatomical assessment for evaluation of the extracellular electrode position within the AL. We used stick insects of the species *Carausius morosus* (Sinéty, 1901), which were bred and kept at Bielefeld University in an artificial 12h:12h light/dark cycle.

**Figure 1:**
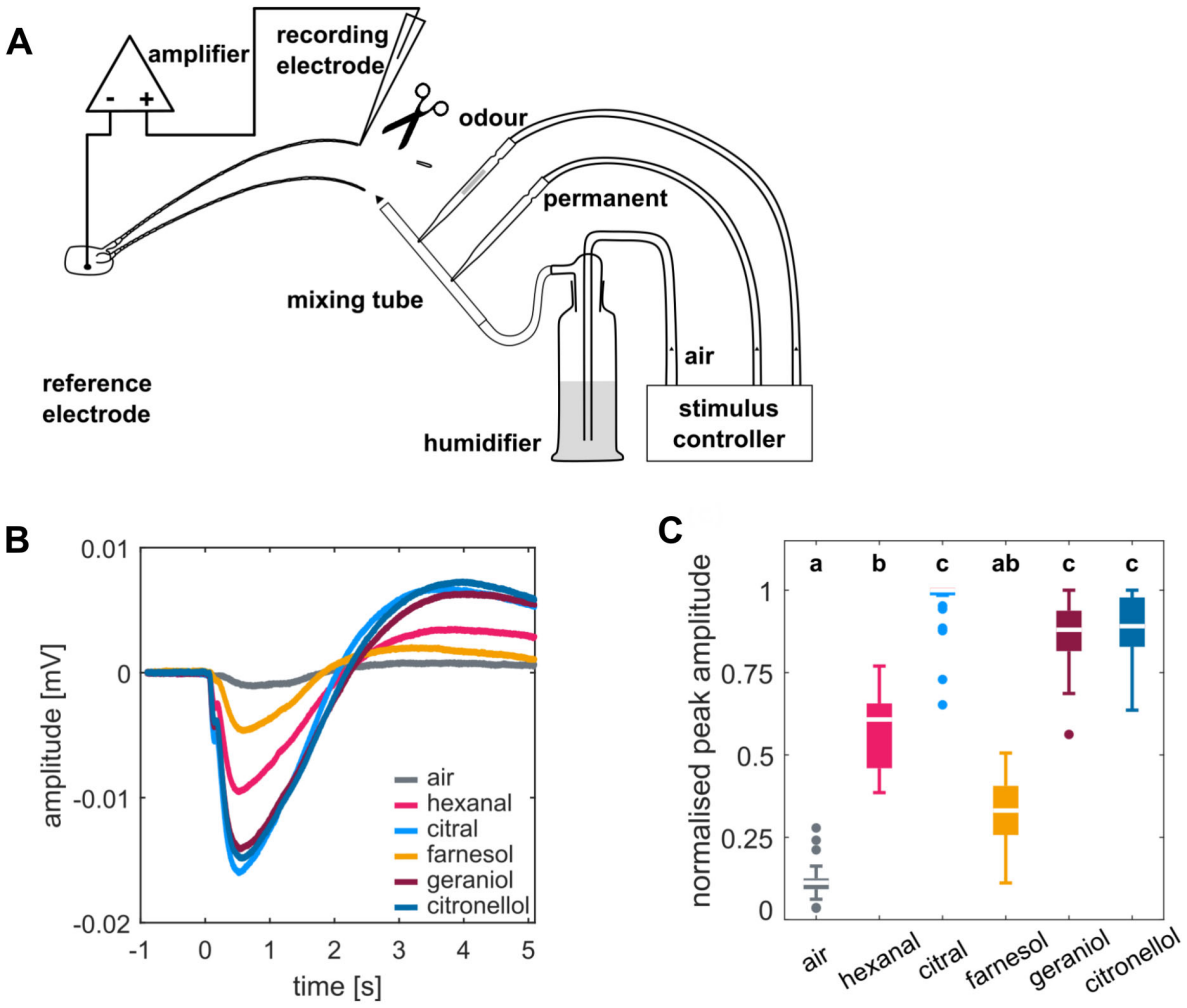
Electroantennograms (EAG) to screen different odours for their ecological relevance. **(A)** Recording setup, with recording electrode at the tip of the last antennomere. A continuous air flow (air) was supplied through a humidifier. For odour stimulation, an additional air flow was injected into the mixing tube. This could be switched from a permanent clean-air stream (reference) to an odour stimulus. **(B)** Average response traces across all animals for five tested odours (cp. colour code) and a clean-air control (grey). Shadings mark the standard error of the mean. **(C)** Odour-dependent normalized peak amplitudes. Box plots comprise n = 25 per-animal means. Letters indicate significantly different amplitudes (Friedman test with & Bonferroni-corrected pos hoc paired comparisons.

**Figure 2:**
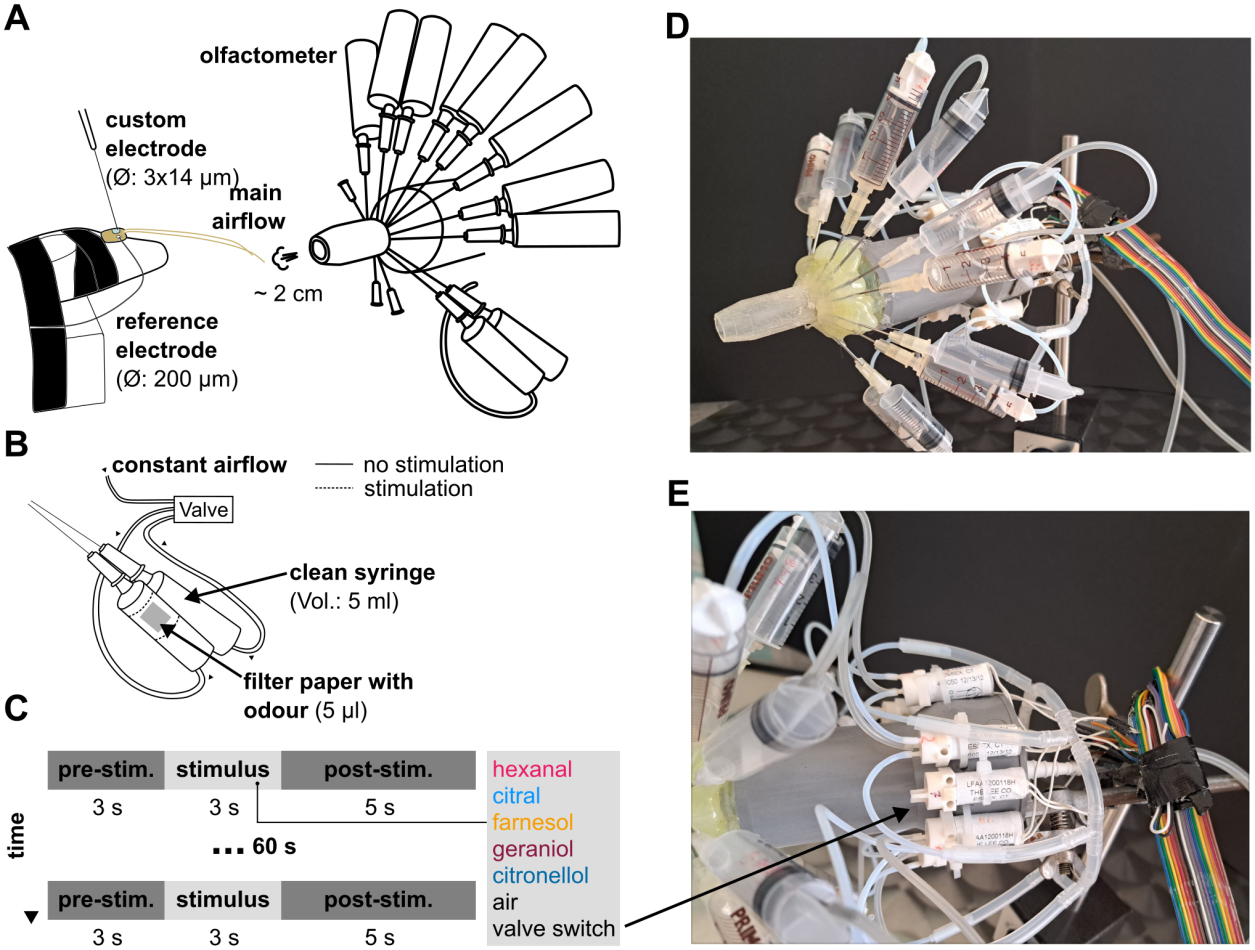
Setup for odour stimulation and experimental procedure for multi-unit recordings. **(A)** Antennal lobe neurons were recorded with a custom-built three-channel wire electrode. Odours were injected in the olfactometer’s main air flow. The nozzle was positioned about 2 cm in front of the antennae. **(B)** Close-up of the syringes used for odour injection. To avoid physical stimulation artefacts, two syringes were used for each odour pathway, one with an empty chamber (clean syringe) and one containing odour (filter paper). The needles of both syringes were injecting into the main air flow. During stimulation the air flow was switched between both chambers via a three-way solenoid valve. The olfactometer was equipped with five odour pathways (5x2 syringes) and one clean-air control (1x2 syringes). An additional solenoid valve not connected to the air system, was mounted between the other valves to control for acoustic or electromagnetic artefacts due to solenoid valve switching [cp. **(D, E)**]. **(C)** Stimulation procedure. Five odour stimuli (cp. colour code) and two controls (air and valve switch) were presented for 3 s in a pseudorandomised manner, each repeated 10 times, with an inter-trial interval of 60 (s) Recordings started 3 s before and lasted until 5 s after stimulation. **(D, E)** Photographs of the custom-built olfactometer.

For both ERG and extracellular multi-unit recordings, adult females were fixed in a custom-made plastic holder with their head immobilised with a small drop of dental wax on either side. Animals were stimulated with a total of five odour components: hexanal (CAS#: 66-25-1), citral (CAS#: 5392-40-5), farnesol (CAS#: 4602-84-0), geraniol (CAS#: 106-24-1), and citronellol (CAS#: 106-22-9), all from Sigma-Aldrich, St. Louis, USA. Hexanal had the highest vapor pressure (1033Pa at 20 °C), followed by geraniol (27Pa at 20 °C), farnesol (13Pa at 20 °C), citronellol (8.6Pa at 20 °C), and citral (4.6Pa at 20 °C), all with a purity >96%. In addition, we presented an air control by leaving the odour chamber empty (**Figures 1**, **2**).

### 2.1 EAG recordings

For conventional EAG recordings, we used a glass capillary electrodes (Hilgenberg GmbH, Malsfeld, Germany) pulled with a Model P-97 micropipette puller (Shutter Instrument Company, Novato, CA, USA) and filled with potassium chloride solution (1 M). The antenna was cut with fine scissors within the most distal antennomere, and the recording electrode was inserted into the opening (**Figure 1A**). Odorants were blown over the remaining antennomeres. The reference electrode (silver wire Ø 25µm) was inserted into the compound eye. Odour compounds were applied using a stimulus controller (Stimulus Controller CS-55, Syntech, Hilversum, The Netherlands), generating a continuous air flow of 1 l/min. The stimulus flow of 0.5 l/min was added via one of two 3 ml glass pipettes that were inserted into the continuous air flow. This resulted in an air speed of 1 m/s at the odour outlet of our mixing tube (**Figure 1**). One pipette connected to an odour chamber equipped with a filter paper containing 5 µl of the pure odorant, the other one connected to an empty chamber. Prior to odour stimulation, the stimulus air flow went through the empty chamber. For providing a timed odour exposure, the air flow switched to the odour chamber for 500 ms, and then back to the empty chamber. This procedure avoids physical stimulation artefacts. Given the stimulus flow of 0.5 l/min and the 500 ms stimulus time an odour containing air volume of about 4 ml was applied. As a control, the odour chamber was equipped with an empty filter paper only. We tested 25 animals, stimulating each with 5 odours and an air control. All stimuli were presented three times in a pseudorandomized order, meaning the order was random, but the same stimulus was not allowed to occur more than twice in a row.

### 2.2 Animal preparation for multi-unit recordings

To get access to the Als, the anterior 1/3 of the dorsal head cuticle was removed. In addition, a small part of the cuticle of the dorsal prothorax was removed and covered with tissue to drain haemolymph. This reduced haemolymph flow around the brain and thus improved visibility during preparation and electrode positioning. Glands, fat body and tracheae on top of the brain were removed, and the cavity then rinsed with Ringer solution (NaCl: 180 mM, KCL: 4 mM, CaCl2∗ 2H2O: 5 mM, MgCl2∗6H2O: 1 mM, HEPES: 10 mM, sucrose: 10 mM, pH: 7.2). Additional Ringer solution was applied whenever necessary to prevent desiccation of the brain. Finally, the neurolemma around both ALs was carefully pierced or partially removed to allow insertion of the electrode.

### 2.3 Multi-unit recordings

Extracellular multi-unit recordings were performed with a custom-made 3-channel wire electrode (described in detail by (Brill et al., 2014)). In brief, three copper wires (coated with polyurethane, 14 μm in diameter, Electrisola, Escholzmatt, Switzerland) were waxed together and connected to an AC pre-amplifier (Headstage-27 Amplifier, Neuralynx, Bozeman, USA). A reference electrode (AG5488 SilverWire, 0.2 mm in diameter, Advent Research Materials) was placed into the right compound eye of the animal. Neural activity was recorded differentially from all three pairwise wire combinations at 30 kHz via a 16-channel recording system (Digital Lynx SX, Neuralynx, Bozeman, USA), and the signal was pre-filtered with a bandpass filter at 200 Hz to 3 kHz and monitored via the Cheetah data acquisition software (Cheetah 5, Neuralynx). The electrode was positioned posterior to the ALs on either side near the medial axis of the animal to record from PNs converging from within the AL at a depth of 130 - 240 μm (corresponding to the m-APT of other insects, as reviewed by (Galizia and Rössler, 2009). For a subset of recordings, the tip of the electrode was coated in the fluorescent dye Alexa Fluor 647 (A20502, Thermo Fischer Scientific GmbH, Dreieich, Germany) for subsequent visualisation of the tip position via confocal microscopy (**Supplementary Figures 1**, **S2**). To avoid misinterpretation of the dye distribution by multiple electrode insertions at the same site, the electrode was inserted only once on each side of the brain. In addition, the opened-up section around the electrode was closed with non-toxic two-component silicone (Kwik-Sil, World Precision Instruments Inc., Sarasota, USA) to fix the electrode relative to the brain, stabilise the brain, and to prevent desiccation of the brain during the long experimental procedure of 90 minutes.

### 2.4 Odour and mechanical stimulation

Olfactory and mechanical stimulation was done by using a custom-made olfactometer as we described earlier (Strube-Bloss et al., 2015). This was modified slightly to produce a pure valve “click” sound. In total, we used seven three-way solenoid valves of the FFAA series (LEE Hydraulische Miniaturkomponnten GmbH, Sulzbach, Germany). Five for the odours, one for the air control, and one for the “valve sound”. For each odour there were two syringes (**Figure 2A**), one with clean air (normally open) and one containing the odour (normally closed, **Figure 2B**). Both syringes were sealed with rubber seal to ensure a confined airflow within the olfactometer. Syringe needles were injected into a constant ‘main’ air stream (**Figures 2A, D**). The flow of this air stream was 1.5 ± 0.1 m/s, as measured at about 2 cm in front of the exit of the olfactometer with a TROTEC TA300 anemometer (Trotec GmbH, Heinsberg, Germany). During stimulation, the three-way valve switched from normally open to normally closed and the airflow was directed through the syringe containing the odour, injecting only about half of the syringe volume into the air stream to avoid concentration gradients (**Figure 2B**). When presenting the air control, the normally closed syringe was empty as well. During the whole experimental procedure, air was sucked out by a ventilation system positioned behind the animal to avoid accumulation of odorants. A total of five odour components (cp. above) and one air control was used. The ‘valve switch’ control was a solenoid valve mounted between the other valves, but disconnected from the tubes (**Figure 2E**). The latter did not result in a change in airflow, but emitted the sound of the ‘valve switch’ (**Figures 2C, E**). For each odour, 5 μl of pure odorant were applied on a piece of filter paper (1cm × 1cm), which was then placed in the corresponding syringe. In total, each stimulus was presented 10 times in a pseudo-randomised order, meaning that the order was random, but the same odour was allowed to follow it self only once. We used an inter-trial interval of 60 s controlled by the ‘trial control’ software from Neuralynx (Bozeman, USA). Each odour stimulation lasted for 3 seconds. Neural activity was recorded from 3 seconds before stimulus onset until 5 seconds after stimulus offset (**Figure 2C**). To estimate the time between stimulus onset (valve switch) and the arrival of odours at the antennae, we calculated the duration of the airflow from the injection needles to the tip of the antennae: Given the distance of 0.05 m between the injection needle and the tip of the antennae and an air speed of 1.5 m/s (see above), the delay between valve switch and odour arrival at the antenna was 33 ms. This delay was always removed when calculating odour induced latencies.

### 2.5 Visualisation of recording position

**Figure 3:**
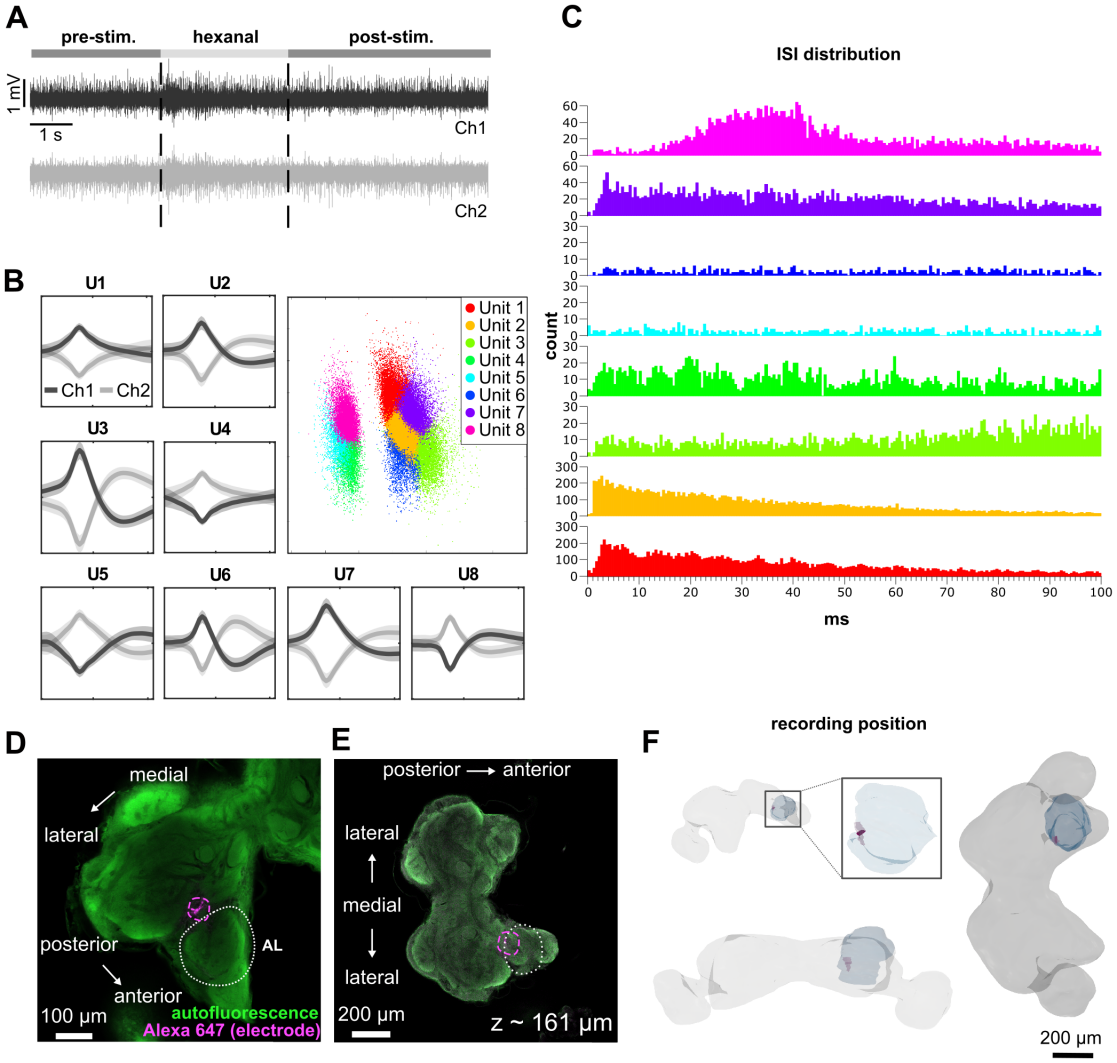
Recordings of AL neurons. **(A)** Raw recording trace from one animal during hexanal stimulation. **(B)** Sorted waveforms from that recording resulting in eight different units (left, ‘U1’ - ‘U8’). Sorting was done with two channels (‘Ch1’ and ‘Ch2’), using the stereo-trode sorting of Spike 2 (cp. Methods). The PCA on the right shows the 1st (x-axis) and 2nd (y-axis) principal components, colour-coded by unit. **(C)** Inter spike interval (ISI) distributions of the units revealed ISIs larger than 1ms. **(D, E)** Confocal brain images after recording. Green: autofluorescence, magenta: fluorescent dye (Alexa 647) from the electrode tip. The white dotted line indicates the location of the AL and the magenta dashed line indicates the dye distribution and thus electrode position. Z-coordinate approximations were calculated from the number of the slice and the distance between slices. Annotated axis directions are based on the animal axis (not the neuraxis). **(F)** 3D reconstruction of **(E)** with the brain surface in grey and the AL in blue. Left: front view and oblique view with magnification of AL. Puncture canal and diffused dye in light magenta. The darker shade of magenta indicates where the dye was most prominent. Right: top view.

In addition to the values obtained from the micromanipulator z-direction in each single recording we exemplary verified the recording position using a visualization protocol in combination with a 3D reconstruction (**Figures 3D-F**; **Supplementary Figure 2** in **Supplementary Material**). After multi-unit recordings, the dye-coated electrode (Alexa Fluor 647) was removed, the brain was dissected in Ringer solution and immediately fixed in FixMix (2% formaldehyde and 2% glutaraldehyde) in phosphate buffered saline (PBS; pH 7.2) at 4 °C for 3–5 days. Afterwards, brains were washed in PBS (5×10 min) at room temperature and then dehydrated in an ascending ethanol series (30%, 50%, 70%, 90%, 95%, 2×100%; 10 min each). For clearing, brains were transferred into methylsalicylate for 10 min and finally mounted in methylsalicylate on a slide with indentation, which was sealed with a cover slip. Samples were then scanned via confocal laser scanning microscopy (Zeiss LSM 780, Carl Zeiss MicroImaging GmbH, Jena, Germany) using a 10× air-immersion objective (EC Plan-Neofluar 10×/0.3, Carl Zeiss MicroImaging GmbH, Jena, Germany) and dual excitation with a DPSS 561–10 and HeNe 633 laser. Brain surface and exterior AL structure as well as electrode position for post-recording samples were reconstructed in 3D, using BioVis3D (Version 3.1, Montevideo, Uruguay). For the electrode position, we highlighted the puncture canal and the most prominent dye position (**Figure 3F**). Real-world scaling of the 3D model is based on the pixel-length of each stack and interval between slices (extracted from meta data). The 3D model from BioVis3D was finally imported in Blender (Blender 3.2) for visualisation. Details and results of histochemistry can be found in the **Supplementary Material** (**Supplementary Figures 1**, **S2**).

### 2.6 Data processing and analyses

#### 2.6.1 EAG data

As the recording baseline before stimulus onset varied between recording situations, we applied a baseline correction to compare between animals by calculating the mean voltage signal 500 ms before stimulus onset and subtracted its value from the complete recording trace. Next, we calculated the mean EAG signal of each animal by averaging the signal of the three repetitions per stimulus (**Figure 1B**). We extracted the odour-dependent maximal amplitudes in each animal and normalized to the odour which evoked the strongest response. To test the odour-dependent normalized peak amplitude distribution for all animals (n=25; **Figure 1c**) we used Friedman’s test with *post-hoc* Wilcoxon’s tests for matched pairs and Bonferroni correction for multiple comparisons (test results in **Supplementary Table S1**).

#### 2.6.2 Spike sorting

Since we recorded multiple channels of extracellular neural activity, comprising spike trains from different neurons, we used a semi-automatic spike-sorting algorithm to extract single-unit activity. Spikes were sorted in Spike2 (Version 8.21, Cambridge Electronic Design, Cambridge, UK) via template-matching based on the different spike waveforms. This was done separately for each recording session. To create waveform templates, threshold-crossing events from either one or two channel(s) (**Figure 3A**) with the best signal-to-noise ratio were used, where the thresholds were manually set and individually chosen for each channel. Spikes (i.e. threshold-crossing events) were assigned to the closest matching template. Afterwards, templates were visually inspected and - if appropriate - similar templates were merged. Quantitatively, we used a principal component analysis (PCA) to assess whether sorted spikes were accurately separated. Considering that we cannot ascertain that spikes of one template come from the same neuron, the term ‘unit’ is better-suited to describe unique waveforms. Examples shown in **Figure 3B** are the mean waveforms and standard errors (shaded area) of eight units from one recording. Since in the shown example recording the clustering of the individual spikes within PCA space (see right of **Figure 3B**) confirms adequate separation of distinct spike events only to a certain level, we included the inter-spike-interval (ISI) distribution of that clusters to ensure ISIs >1ms, which is the refractory period of a neuron (**Figure 3C**). Lastly, event-based time stamps of individual units and stimuli were exported to MATLAB R2019a (The MathWorks, Inc., Natick, USA) for further analysis. In total we extracted 118 units out of 24 animals.

#### 2.6.3 Response detection

**Figure 4:**
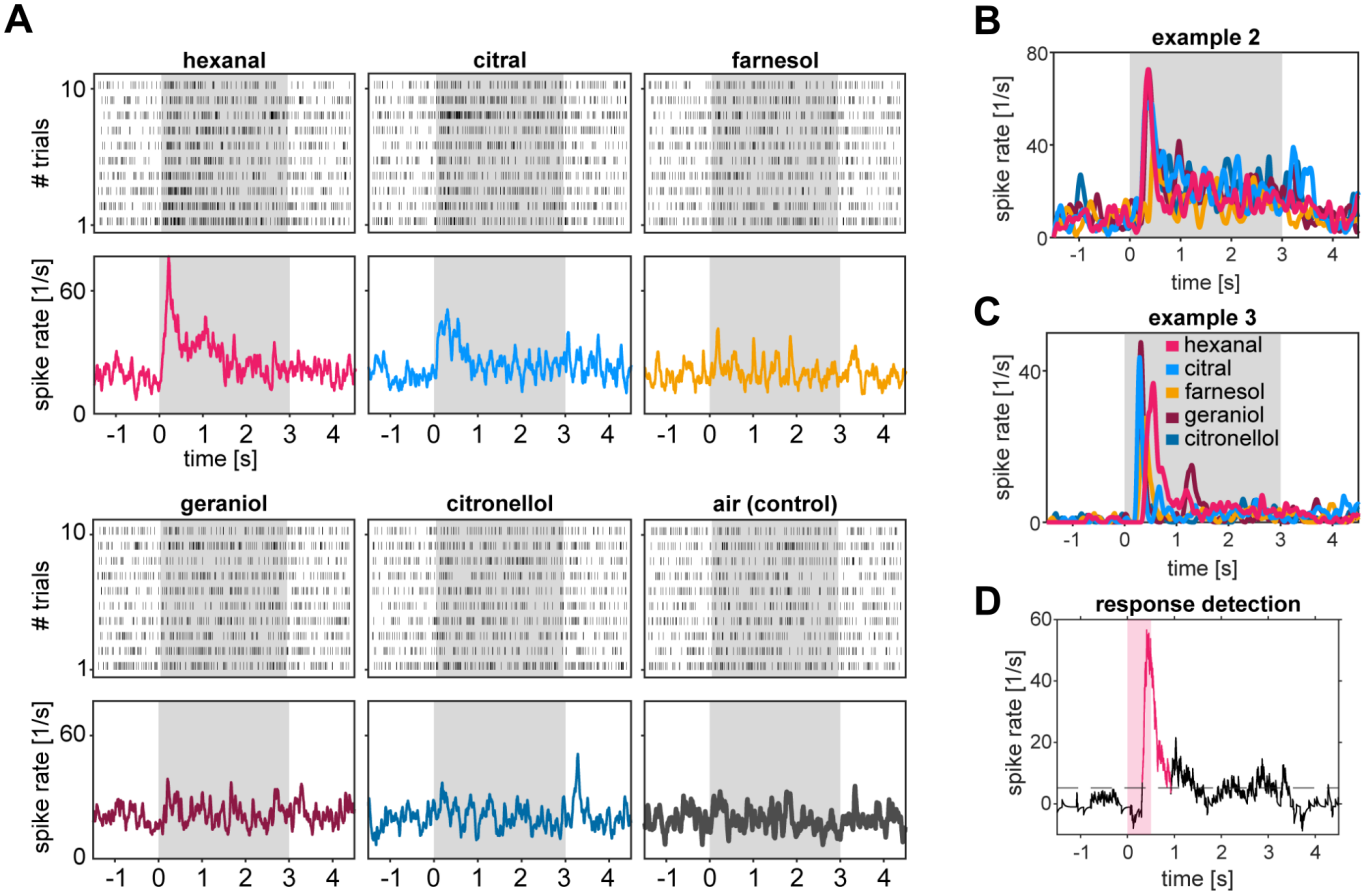
Example units and response detection. **(A)** Peri-stimulus time histogram (PSTH) of a single unit (same as U1 in **Figure 3B**) during stimulation. Top plots show single spikes (black lines) for each trial over time, and bottom plots show spike rates averaged across the ten trials before baseline correction. Stimulation time is indicated by grey shading. Stimulus onset was at time zero. **(B, C)** Averaged spike rates of two example units, both showing pronounced and distinct responses to the different odour stimuli (cp. colour code). **(D)** To decide whether or not a unit responded to a stimulus, we compared the averaged firing rates 1500 ms before and 500 ms during the odour stimulation, setting a threshold at 3 times the standard deviation (dashed line).

To include only units that respond to the stimulation, we first applied a response detection. For each stimulus and unit, we calculated the average spike rate of the ten stimulus repetitions with an exponential kernel estimation in ms resolution (Nawrot et al., 1999), and corrected the baseline by subtracting the mean spike rate 1500 ms before stimulus onset from the whole trace of the firing rate. Based on the resulting stimulus-induced spike rates, we set the detection threshold of a response to ≥3× the standard deviation (3×SD) of the spike rate during 1500 ms before stimulus onset (cp. **Figure 4D**). We looked at potential responses up to 500 ms after stimulus onset. For further response related analysis, we only used a subset of 96 units that responded to at least one stimulus.

#### 2.6.4 Response latency

**Figure 5:**
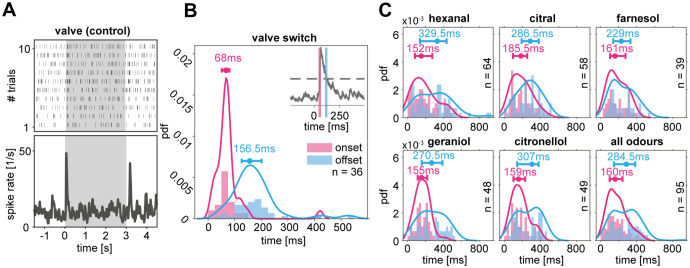
Response latency differences in mechanosensory and olfactory driven units. **(A)** Example of a valve switch response. **(B)** Probability density function estimate of threshold crossings of 36 units for rising (magenta; onset) and falling slopes (cyan; offset). An example of a single-unit response is shown in the inset (dashed line shows 3×SD threshold). Valve-switch-induced responses start with a median delay of 68 ms and last until 156.5 ms (symbols and error bars show medians and interquartile ranges). **(C)** Response threshold crossings of units responding to the odour stimulation. Most units responded to hexanal (n=64) with a median latency of 152 ms and median offset at 329.5 ms (medians and interquartile ranges), followed by citral (n=58), citronellol (n=49), geraniol (n=48), and farnesol (n=39). Same plot details as for hexanal. Taking all odour-induced firing rates together (lower right), odour responses started 160 ms and ended 284.5 ms after odour onset (symbols and error bars show medians and interquartile ranges).

**Figure 6:**
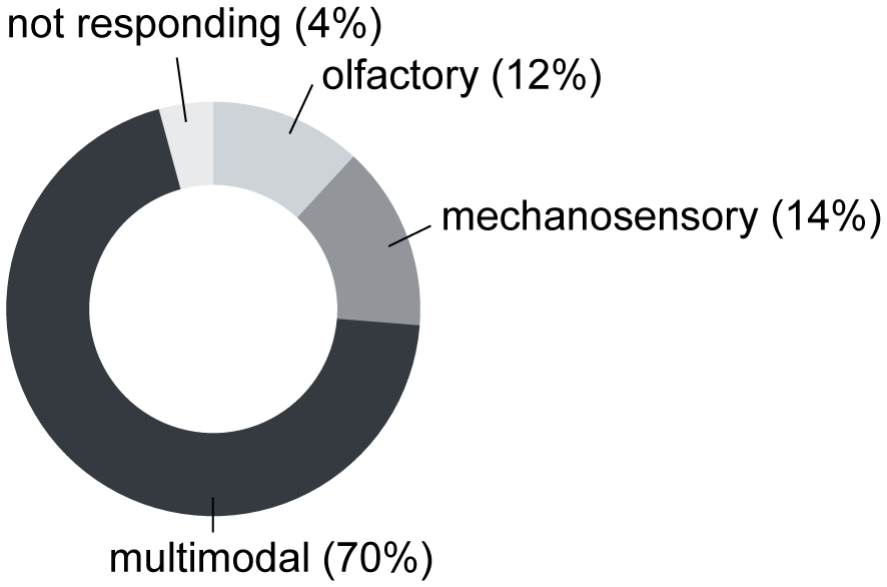
Olfactory, mechanosensory and bimodal AL- neurons. Portion of all 118 recorded units responding during the valve switch only (detection window 0–157 ms, mechanosensory), the olfactory stimulus only (detection window 157–500 ms, olfactory) or to both types of stimuli (both detection windows). A small group of neurons (4%) did not respond to any stimulus.

To analyse temporal dynamics of responding units, we looked at the timing of response onsets and offsets. We defined the onset as the first event at which the spike rate estimation (i.e. the rising phase of the response) exceeded the detection threshold (3×SD). The offset was set to the first downward crossing of the threshold during the descending phase. We did not implement response detection for inhibitory responses, since they appeared rather rarely. The onsets and offsets were determined for all responded units and all odour stimuli, as well as for a subset of 36 units that were tested with the valve switch control and responded to it (**Figure 5B**). To compare the dynamics of olfactory responses to dynamics of responses to the valve switch, we compared each distribution of olfactory response onsets and offsets to the distribution of valve response onsets and offsets, respectively, using a two-sample Kolmogorov-Smirnov test with Bonferroni correction for multiple testing (α_corr_ = 0.05/5 = 0.01; test results in **Supplementary Table S2**). The Proportion of units responding to olfactory, mechanosensory and both of the modalities are given in **Figure 6**.

#### 2.6.5 Odour separation within the recorded population

**Figure 7:**
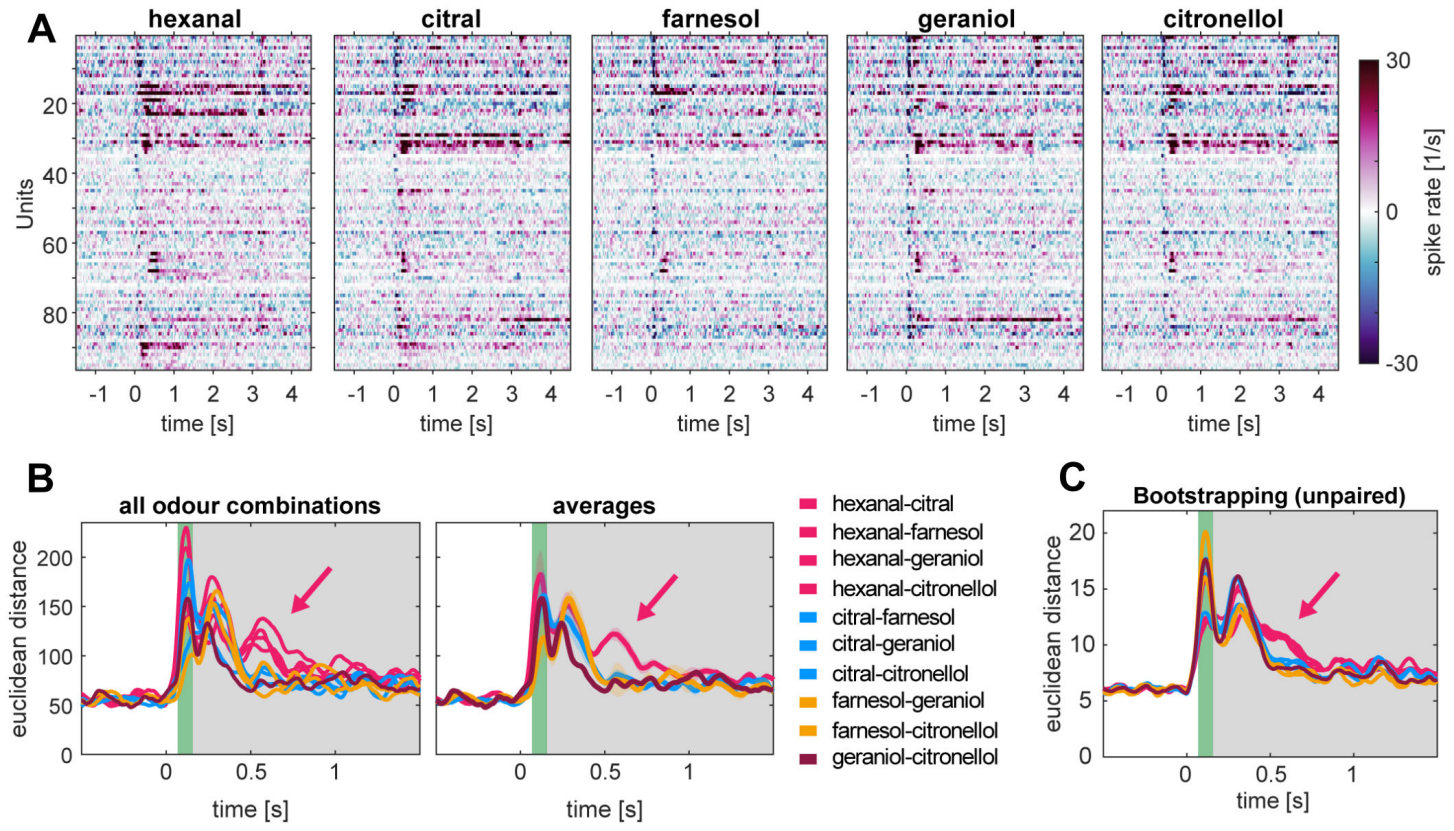
Population response separates hexanal. **(A)** Population matrices with averaged firing rates of N = 96 units from n = 24 animals that showed olfactory responses. Matrix rows are units, columns are average spike rates per unit in ms. The spike rate is indicated by the colour gradient to the right. Stimulation lasted for three seconds and started at 0 (s) **(B)** Time courses of pair-wise Euclidean distances (ED in 1/s) between all combinations of the population response matrices (left) and averages (coloured shaded area: SEM) of each combination group (right). For visualisation, individual traces were smoothed with a running-average kernel with a width of 75 ms. The green area indicates timing of valve response and the grey area indicates odour stimulation. **(C)** Bootstrapped (unpaired) Euclidean distances retain odour separation.

Separately for each stimulus, we constructed n-dimensional population vectors for ensembles of n units including all units which responded to at least one odour (**Figure 7A**). This was done for each point in time (v(t)), using the mean spike rate. Pair-wise time-resolved Euclidean Distances (EDs) (L2-Norm) were determined for each stimulus (odour) combination. For two stimuli, *a* and *b*, the distance *d* at time *t* was calculated as follows (**Figure 7B**):

(1) $$\text{d}(t)=\sqrt{\sum_{i=1}^{n}\;{\left(v_{i}^{a}(t)-v_{i}^{b}(t)\right)}^{2}}$$

**Figure 8:**
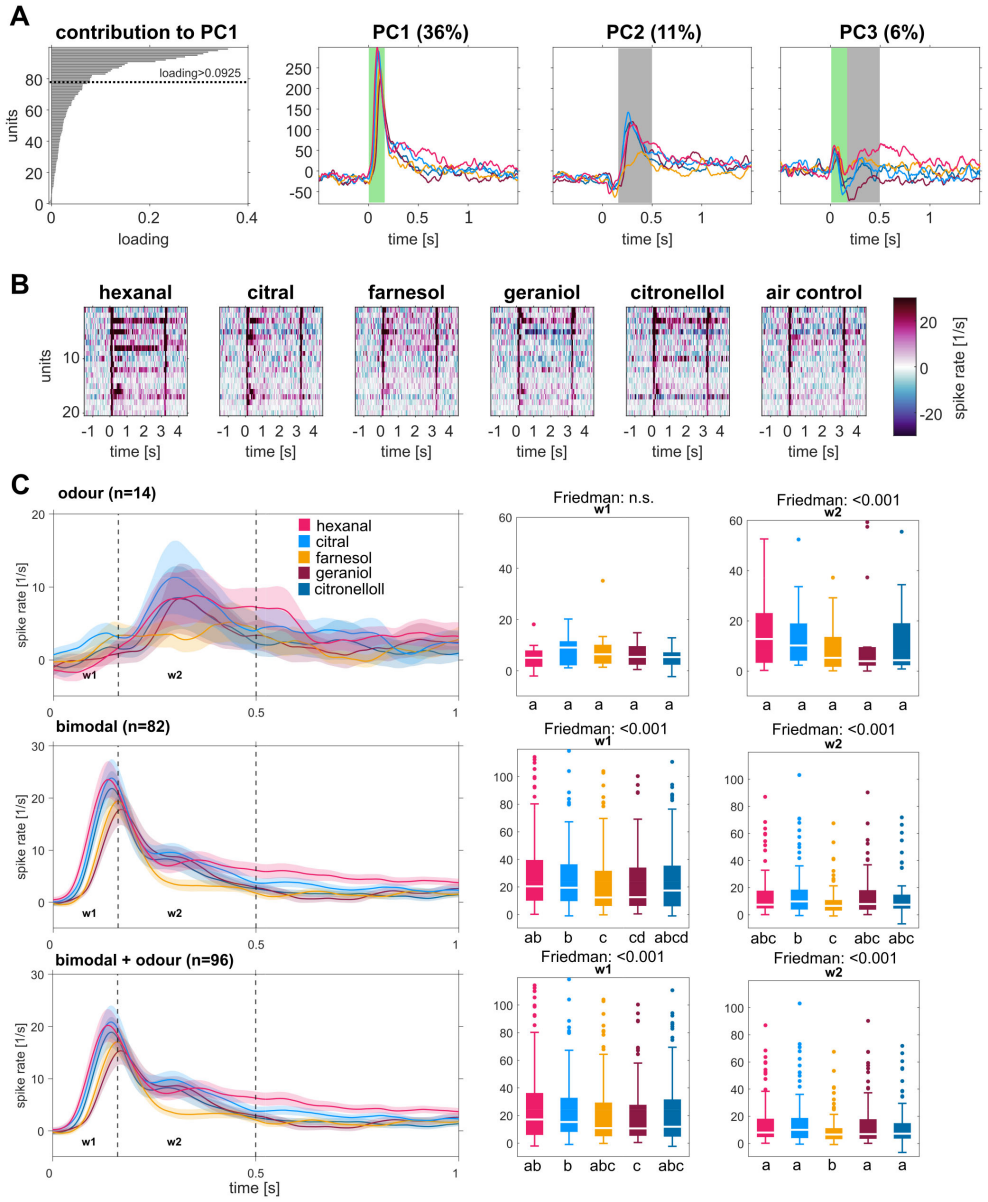
Principal component analysis extracts mechanosensory and olfactory responses. **(A)** Factor loadings of all bimodal and olfactory-sensitive units for PC1 (most left panel). Main contributing units were defined as units with a loading > 0.0925 (n=20). PCs 1–3 explained 36%,11%, and 6%, respectively of the total variance. The time courses of each component, colour-coded by stimulus (sp. (c)), are shown in the three subplots on the right. PC1 indicates the separation of mechanosensory activity during the valve switch (0–157 ms, green), whereas PC2 extracts activity during the odour response window (157-500ms, grey). PC3 contrasted both. **(B)** Population vectors of most contributing units (from a). **(C)** Odour-dependent averaged spike rates (cp. colour code) of unimodal odour sensitive units (n=14; top), bimodal units (n=82; middle), and both of the odour activated groups (n=96; bottom). Dashed lines mark the time windows used for comparative analysis of mechanosensory activity (0–157 ms=w1) and odour-dependent activity, starting approximately (157–500 ms=w2) after stimulus onset. Boxplots on the right illustrate the maximal spike rate distribution of all units in the respective time window. To cover the peak of the odour induced activity we started at 250 ms after odour onset (time 0 ms). Significance levels of a Friedman test are indicated above, and significance levels of *post-hoc* multiple-comparison Wilcoxon signed-rank tests with Bonferroni-corrected alpha values are below. Different letters indicate significant differences (cp. **Supplementary Table S3**).

To further test the robustness of the population activity, we calculated ‘bootstrapped’ EDs from arbitrary pairs of responding units. To do so, we randomly sampled two units at a time and calculated the ED with 10.000 repetitions per odour combination (**Figure 7C**). Furthermore, we visualized the data using a PCA, where points in time were used as observations and units as variables (and thus dimensions of the original component space), separating mechanosensory and olfactory induced population activity. Units with factor loadings of > 0.0925 illustrate the main contributing units (n = 20; **Figures 8A, B**).

We compared firing rates of odour sensitive and bimodal units within the mechanosensory and the odour response window for the different stimuli (**Figure 8C**). For visualization, individual traces were filtered with a running average kernel with a width of 75 ms. We used a Friedman test (per time-window) to test for differences in mean firing rates and *post-hoc* Wilcoxon signed rank tests with an alpha value adjustment after Bonferroni (α_corr_ = 0.05/10 = 0.005), since we tested every other odour against each other (**Figure 8C**; test results in **Supplementary Table S3**).

## 3 Results

Using extracellular multi-unit recordings in combination with spike-sorting we were able to extract 96 potential AL-neurons (single units) from 24 animals that responded to at least one of our olfactory and non-olfactory stimuli. To verify the ecological relevance of the odour component selection, we first recorded EAGs in another 25 animals and stimulated with hexanal, citral, farnesol, geraniol and citronellol (**Figures 1A–C**). Although hexanal evoked the most prominent separation at the AL level (see below), geraniol and citronellol dominated the EAG response (**Figure 1C**). In addition to odour-induced activity, we found evidence for mechanically induced activity within the AL in response to solenoid valve “clicks” which occurred when switching to the odour stimulus.

### 3.1 Olfactory and mechanosensory AL-activity

Olfactory response profiles generally differed across the recorded units. For example, unit 1 in **Figure 4a** (same as U1 in **Figure 3B**) shows a clear response to hexanal and citral, as reflected by a phasic-tonic increase in spike rate, but neither to any of the other odours, nor to the air control (**Figure 4A**). Other units showed responses to all odours, although with varying amplitude (**Figure 4B**). Yet other units responded to all odours, but with different temporal dynamics, where the response to hexanal appeared at a later phase compared to responses to other odours (**Figure 4C**). “Responding units” were defined according to a response detection threshold, including only units whose firing rate increased after stimulus onset by at least three standard deviations of the resting activity (**Figure 4D**). Application of this response detection threshold not only revealed odour-induced activity, it also reliably extracted salient response peaks at both the onset and offset of the clean-air control. To ensure that this type of responses was neither driven by chemical contamination of the olfactometers’ hose system, nor a physical artefact, we added an additional solenoid valve which was disconnected from the hose system to test for olfactometer-independent responses (valve control, **Figure 2E**). This way, we could exclude artefacts such as responses to small air pressure changes during the switch between the empty and the odour-loaded syringe (see **Figure 2B**). Indeed, this valve control stimulus reliably induced short phasic responses in 36 units (**Figures 5A, B**).

### 3.2 A rapid mechanosensory response may lead olfactory responses

The responses to the valve control stimulus (**Figure 5A**) were very transient. We attribute them to be induced by a ‘click’ sound which occurred when the valves switched the airflow between each pair of syringes to initiate or terminate the odour stimulation. As we can exclude electromagnetically induced activity and other artefacts (cp. discussion), we conclude the presence of a subpopulation of AL neurons that shows mechanosensory sensitivity to airborne vibrations (subsequently referred to as ‘valve responses’). Since the olfactory responses can only occur subsequently to the valve switch, we needed to distinguish between response components induced by the valve switch or odour stimulus, respectively. To do so, we analysed the temporal dynamics in more detail, delimiting the timing of the transient valve responses. The onset and offset of the valve response peak was determined as the time point at which the firing rate of a unit exceeded or fell below the response threshold, respectively (**Figure 4D**). This analysis revealed that ‘valve responses’ had an early onset and offset (**Figure 5B**), whereas olfactory responses had a later onset and offset (**Figure 5C**). In fact, the median onset of olfactory responses (160 ms) was close to the median offset of the valve response (156.5 ms). We performed a two-sample Kolmogorov-Smirnov test to analyse whether values from valve responses and values from olfactory responses had the same underlying distribution. This was tested separately for each odour and for onset and offset values. All onset and offset values of olfactory responses were significantly different from those of the ‘valve responses’ (**Supplementary Table S2**). Taken together, stimulus sequence, i.e. switching the valve followed by blowing the odour, was reflected by the sequential response dynamics of the recorded neurons. Accordingly, we adjusted the response detection windows for mechanically induced activity between 0 to 157 ms, and for odour induced responses between 157 ms – 500 ms after stimulus onset.

### 3.3 Olfactory, mechanosensory and combinatorial (multimodal) AL neuron subpopulations

The largest number of AL-neurons were sensitive to hexanal (n=64; **Figure 5B**). This was closely followed by citral (n=58; **Figure 5B**), whereas the fewest neurons responded to farnesol (n=39; **Figure 5B**). Note that this includes all units that responded during the odour detection window (157 to 500 ms). To estimate the proportion of units sensitive to the mechanosensory component produced by the ‘valve switch’, we counted all units that responded during the early response window (0 to 157 ms). This not only includes units sensitive to the ‘valve switch’, but may also include bimodal neurons with persistent activity into the subsequent odour response window. **Figure 6** shows the proportion of units responding during none, one or both response windows. In summary, the largest portion of units (70%) responded during both the early and the odour response window and, therefore, comprised putatively bimodal units. Further 26% of units responded during one of both response windows only. As we can rule out mechanosensory-induced activity induced by the valve switch during the odour response window, but not rule out olfactory-induced activity during the early response window, these subsets of units were unimodal olfactory (12%) and putatively unimodal mechanosensory (14%) units, respectively.

### 3.4 Separation of hexanal by odour-sensitive AL-neurons

After adjusting the response detection window, we characterized units showing olfactory responses, i.e. units that showed a response to at least one odour stimulus. In total we extracted 96 odour-sensitive units in 24 animal preparations. This included units that responded to odour only and units that responded bimodally to both odour and valve click. We used the 96 individual mean response time courses to construct time-varying population response vectors (**Figure 7A**). Predominantly, the recorded units showed either an excitatory response or no response, while inhibitory responses were observed rather rarely. Note that **Figure 7A** shows spike rate changes relative to baseline activity, such that stimulus-independent spike rates fluctuated around zero. Furthermore, temporal dynamics of responses differed across units. Apart from the late response onsets mentioned earlier, some units showed phasic responses, and some showed longer-lasting phasic-tonic responses. The strongest responses appeared to be induced by hexanal and citral, whereas farnesol caused the weakest changes in activity. To analyse the extent to which odour-induced activity patterns differed across the population of odour-sensitive AL neurons, we calculated time-resolved pair-wise Euclidean distances (ED) (Equation 1) between all population vector pairs (**Figure 7B**). Shortly after stimulus onset, there were three peaks; the first was extremely transient and can be related to the valve response time window (0-157ms). Most likely, this peak was driven by bimodal AL neurons, i.e. neurons sensitive to odour and mechanosensitive stimulation. Thus, the ‘valve switch’ already induced an odour-dependent neural activity. Since each odour was switched by its individual solenoid valve, we analysed the audio recordings of the valves (**Supplementary Figure 3**). The power spectra revealed a valve-specific composition of high frequencies in the range of 1-20kHz.

The second and third peaks decreased more slowly over time, spanning the entire olfactory response time window. While olfactory separation was inconsistent as reflected by a large overlap of the distance measures during the second peak, this changed as the second peak subsided, with a consistent increase of all EDs to hexanal, peaking approximately 500 ms after stimulus onset (**Figures 7B**, left). This consistency is underscored by distinct separation of the average trace from all hexanal-containing pairs (**Figures 7B**, right). Moreover, this separation persisted even after removing the pairing by means of randomly sampled pairs of units (bootstrapping), where the third peak remained as a slowly decaying shoulder between 400 and 750 ms after stimulus onset (**Figure 7C**). Thus, AL ensemble activity revealed a distinct separation pattern for hexanal compared to the separation between the other odour couples tested.

### 3.5 Unimodal and bimodal units are responsible for odour separation

To further focus our analysis on units responsible for the distinct hexanal response, we performed a PCA and concentrated on the first three principal components (PC1-3; **Figure 8A**). The time course of PC1 (36% of variance explained) reflected mechanosensory activity during the valve switch (0–157 ms) before the odour response window (157-500ms), which was extracted by PC2 (11% of variance explained). PC3 contrasted both time windows. The subset of units which contributed most to the variation along PC1 were shown in more detail in **Figure 8B** illustrating that it was a combination of unimodal and bimodal units. Some units showed a phasic response to the onset (mechanosensory) only, whereas other units showed the same phasic activity followed by tonic activity during the odour presentation, which seemed to be most pronounced for hexanal. To further analyse what unimodal and bimodal units might contribute to the odour separation, we calculated odour-dependent averaged spike rates of unimodal odour-sensitive units (n=14) and bimodal units (n=82; **Figure 8**). Interestingly, unimodal odour-sensitive units alone did not show an odour-dependent maximal rate distribution during the odour response window (157–500 ms). However, the population of bimodal units showed significant effects in both the mechanosensory response window (0–157 ms) and the odour response window (157–500 ms). Combing both unimodal and bimodal units in one analysis (**Figures 8C**, bottom) revealed that hexanal, citral, and geraniol evoked the highest activity to the same extent, whereas citronellol and farnesol, which were not significantly differentiated from each other, evoked a significantly lower maximal response rate (Friedman Test, p<0.001; followed by Wilcoxon signed rank test with an alpha value correction after Bonferroni (αcorr = 0.05/10 = 0.005)). Statistical details of all units and windows are given in **Supplementary Table S3**.

### 3.6 Recording position and anatomy

Since our aim was to record from neurons in the deutocerebral output region of the AL, and the deutocerebrum contains both olfactory and mechanosensory processing neuropiles, we needed to confirm the recording position within the deutocerebrum. To do so, we regularly dissected example brains, which were subsequently processed to be scanned via confocal laser scanning microscopy. Although the extracellular recording did not allow identification of single neurons, visualization and 3D reconstruction of the electrode position confirmed that we recorded from the output region of the AL. More specifically, recording positions were always posterior within the dorsal AL rim, and medio-lateral with reference to the whole brain (**Figures 3D–F**; for further examples see **Supplementary Figure 2** of the **Supplementary Material**). This corresponds to the region where evidence from other insect species suggests AL projection neurons to bundle to form the AL tract (Galizia and Rössler, 2009). Visualization and reconstruction of the whole stick insect brain revealed the overall dimensions of its AL, showing a dorso-ventral elongation of about 300 µm (**Supplementary Figure 1** in **Supplementary Material**). During our recordings, the depth of the electrode tip ranged from 133 μm to 240 μm (197.13 ± 32.89 μm, mean ± standard deviation and, thus, lay within this dorso-ventral dimension of the AL. We conclude that all units recorded in this study were part of the AL network, indicating that the AL of stick insects processed both olfactory and mechanosensory afferent information.

## 4 Discussion

### 4.1 The AL network separates odours of behavioural relevance

When it comes to odour processing, the stick insect *C. morosus* is rather understudied. To investigate how olfactory information is processed at different stages within the stick insect’s olfactory system, we compared recordings of peripheral bulk receptor activity by means of EAG recordings in intact stick insects with odour separation at the next processing level, the AL.

Since all tested odorants evoked a significant EAG signal, we concluded that these compounds were detected by antennal receptors and are therefore ecologically relevant for stick insects. As we used pure odour components, effects of dilution media and potential chemical reactions can be neglected. However, different vapour pressures affect the volatility and, consequently, the number of molecules in the surrounding air, which might impact the population of excited OSNs. Additionally, the number of OSNs that are sensitive to a particular odorant might differ, further affecting EAG signal strength. Our data suggests both. Among the less volatile components, the group of citral (vapour pressure: 4.6 Pa at 20 °C), geraniol (vapour pressure: 27 Pa at 20 °C), and cironellol (vapour pressure: 8.6 Pa at 20 °C) evoked the strongest EAG responses, and differed from the response to farnesol (vapour pressure: 13 Pa at 20 °C), despite farnesol having a similarly low vapour pressure. This significant difference might be due to fewer farnesol-sensitive OSNs, suggesting that farnesol may have lower behavioural importance. Since farnesol is an odour component of many flowers and also part of the recruitment pheromone of bumblebees (Dornhaus and Chittka, 2001; Dornhaus et al., 2003; Granero et al., 2005) where it is processed at the AL level (Strube-Bloss et al., 2015), it may not play a role for stick insects. On the other hand, highly volatile hexanal (vapour pressure: 1033 Pa at 20 °C) was expected to reach a higher molecule concentration in the air than the other odorants, yet elicited a significantly weaker EAG response compared to first three mentioned above (**Figure 1C**). Thus, EAG signal strength neither mirrored vapour pressure of the molecule, nor did it relate to the putative behavioural relevance of hexanal to an herbivore.

Comparing EAG and AL response levels suggested a form of odour tuning, starting within the AL-network, as the odour response profiles showed a different ranking. Hence, different odours might vary in their behavioural relevance for these animals. This is reminiscent of an earlier result in bumblebees, where farnesol as a component of the bumblebees’ recruitment pheromone evoked low activity at the EAG level, but increased separation by the AL neural network (Strube-Bloss et al., 2015). Comparing the EAG signal (**Figures 1B, C**) with the AL network separation at the population level (**Figure 7**) revealed the same effect for hexanal. Hexanal evoked the second lowest EAG response, yet at the AL output level, ensemble activity led to a distinct separation of hexanal from other tested odour components during the late odour response window (~500 ms). Thus, the representation of odours was adjusted from the peripheral level to the next processing stage of the olfactory pathway, the AL, where the neural representation of hexanal was significantly prolonged. Hexanal belongs to the group of green-leaf volatiles. It is a characteristic component of various plants and is likely important for herbivores, such as stick insects, in the context of foraging. Hexanal is released when leaves are damaged and has been suggested to act as a direct and indirect defence mechanism of plants against herbivores (Arimura et al., 2005). Judging from fieldwork sightings (personal observations, e.g. on https://www.inaturalist.org/), *Carausius morosus* is often found to feed on plants of the genus *Rubus* (e.g., blackberry, bramble). While we have no data on whether blackberry leaves contain hexanal, the blackberry fruit does (Qian and Wang, 2005). Therefore, the release of hexanal may serve as an olfactory cue for stick insects. Whether it is as attractive as expected for an appetitive cue has yet to be shown behaviourally.

### 4.2 Delayed separation of hexanal

We found that the recorded olfactory AL neurons of stick insects show phasic and phasic-tonic, excitatory responses shortly after stimulus onset. These firing patterns match those of AL neurons in bees (Strube-Bloss et al., 2015; Krofczik et al., 2008; Abel et al., 2001; Müller et al., 2002; Galizia and Kimmerle, 2004), moths (Namiki and Kanzaki, 2008), flies (Wilson et al., 2004), and locusts (Anton and Hansson, 1996). Since we rarely observed inhibition (i.e. a reduction of spike rate), response detection was applied to excitatory responding units only. The recorded units revealed a broad response profile and low selectivity (**Figure 8C**), which has already been described in some PNs of *Drosophila melanogaster* (Wilson et al., 2004; Bhandawat et al., 2007). The broad response profile as observed here (**Figure 8C**) might be based on lateral excitation from excitatory LNs, as described in other insect species (Husch et al., 2009; Reisenman et al., 2011). In comparison, PNs of honeybees are usually more selective (Krofczik et al., 2008; Strube-Bloss et al., 2012), because of sharpening of glomerular activation patterns by inhibiting weakly activated glomeruli (Sachse and Galizia, 2002). Among the more than 400 LNs found in the ALs of the fruit fly, which fall into 74 types across 25 anatomical groups, there are at least 3 types that based on functional neuroanatomic studies are known to be inhibitory (Schlegel et al., 2021; Sizemore et al., 2023; Sizemore and Dacks, 2016). Within this broad diversity of LN types, at least two types of inhibitory LNs shape PN odour responses. While they are both odour and glomerulus-specific, one type could act during the early response phase (via GABA_A_ receptors), whereas the other during the later phase at 1.5 to 2.5 s after stimulus onset, via GABA_B_ receptors (Wilson and Laurent, 2005). Although this is purely speculative, the initial broad response profile in our data might be explained by excitatory LNs, whereas the sustained increased activity in the response to only hexanal might be explained by inhibitory LNs, which act on a delayed timescale. Thus, glomeruli that are activated by hexanal would maintain a high activity profile, whereas glomeruli that are activated by the remaining odour components would be inhibited (shut down) in a secondary processing phase. And while PN responses of bees and flies usually emphasize the onset of a stimulus, it was shown for locusts that the representation of an odour becomes more distinct with increasing time (Stopfer et al., 2003), which could explain why we see the separation of hexanal in the Euclidean Distances (**Figure 7**) only during a rather late phase of the response. Since we used pure odorants, the observed late separation might also be due to the high concentrations presented, as an increase in odour concentration is accompanied by an expansion in spatial distribution, temporal complexity, and duration (Daly et al., 2015). Thus, future experiments should include different odour concentrations within their experimental design.

### 4.3 Mechanosensitive AL neurons

Besides establishing extracellular multi-unit-recordings in the AL neurons in *C. morosus*, which confirm general coding principles along the olfactory pathway of insects, our most striking result is the additional mechanical activation of AL neurons at this early processing level, indicating the presence of mechanosensitive interneurons within the AL. By implementing a control stimulus in which the solenoid valve was not connected to any hose (**Figure 2E**), we were able to exclude physical artefacts originating from the airflow switching between empty and filled odour chambers. Although some insects, like ants, can sense magnetic fields (Fleischmann et al., 2018; Grob et al., 2024a, b), which might be produced by electrical switching of solenoid valves, we argue that they rather responded to the sound produced by switching valves. The latter was supported by analysing the audio recordings of the seven solenoid valves used, each producing a valve-specific frequency composition in the range of 1-20kHz (**Supplementary Figure 3**). However, to analyse and characterize the mechanosensory component in more detail, we are currently testing a set of single frequencies in an ongoing experiment. Indeed, when testing these putative mechanosensitive neurons with defined acoustical stimuli, we found a complex, stimulus-dependent, combinatorial response pattern of excitatory and inhibitory response components within the same neuron (**Supplementary Figure 4**). This supports the idea of parallel processing of olfactory and mechanosensory information within the AL circuitry. The latter has been shown in the honeybee, where clean air pulses generate complex patterns of wind-speed-dependent glomerular activity, which is superimposed with olfactory-induced activity during bimodal stimulation (Tiraboschi et al., 2021). Similarly, in moths, the sound of a predatory bat sensitize olfactory neurons at the AL level (Anton et al., 2011), while in cockroaches AL neurons (LNs and PNs) respond to both modalities (Zeiner and Tichy, 1998), showing bimodal combinatorial effect that leads to an enhanced or depressed response compared to unimodal stimulation. In agreement with these results, here we found mechanosensitive AL neurons to be common in *C. morosus*, including a large proportion of bimodal neurons. This kind of mechanosensory code within the olfactory system has also been found in mammals (Grosmaitre et al., 2007; Carey et al., 2009) and will be part of our future investigations using the stick insect as a promising model to explore olfactory and mechanosensory integration and related effects on motor control.

### 4.4 Origin of the mechanosensory response

A potential source for the mechanosensory signal could be located in the olfactory sensilla hairs on the antenna surface, as discussed by Tiraboschi and colleagues (Tiraboschi et al., 2021). Their data suggest that mechanical changes in sensilla position, motion, or shape might activate AL neurons responsible for modulating the glomerular pattern. Another candidate mechanoreceptor could be a chordotonal organ. Chordotonal organs can sense vibrations (Field and Matheson, 1998), and Johnston’s organ (JO), a specialised chordotonal organ of the pterygote antenna (Dürr et al., 2022), detects acoustic signals (Lai et al., 2012). In stick insects, JO sensitivity to acoustic signals has not yet been demonstrated, though by homology of function it could be the source of the recorded mechanosensory response. In *Drosophila melanogaster* and the ant *Cataglyphis nodus*, afferents of the JO bypass the AL and project into the antennal mechanosensory and motor center (AMMC) instead (Lai et al., 2012; Grob et al., 2021). In *C. morosus* bulk stains of antennal mechanosensory afferents have not revealed terminals in the AL (Goldammer and Dürr, 2018) and potential second-order projections into the AL has not been investigated. Here, we can exclude that we recorded neurons within the AMMC, since we controlled for electrode depth and position. The post-recording confocal scans confirmed that the electrode tip was never deeper than the total AL dilatation (**Figures 3D–F**; **Supplementary Figures 1**, **S2** in **Supplementary Material**). Our estimated AL elongation from the synapsin scans (**Supplementary Figure 1**) was roughly 300 μm, whereas the maximal electrode depth was 240 μm and the average was 197.13 ± 32.89 μm (compare with examples in **Supplementary Figure 2** in **Supplementary Material**). Since the AMMC in stick insects is located ventrally, below the AL (owing to the neuraxis being tilted backwards, Goldammer and Dürr, 2018), our recording positions were far away from the AMMC. Moreover, owing to the applied differentiation between all pair-wise combinations of the single electrode wires, we are certain that the source of the measured spike activity was very close to the electrode tip and, therefore, the reported olfactory and mechanosensory responses reflects AL neural activity.

## 5 Conclusion

Our study provides significant insights into the processing of olfactory information within the nervous system of the stick insect, *Carausius morosus*. By comparing peripheral receptor activity with AL processing, we observed a distinct tuning for odorants, suggesting an adaptive olfactory processing mechanism, possibly linked to the behavioural significance of hexanal as a foraging cue. Moreover, our findings highlight the presence of mechanosensitive neurons within the AL, indicating parallel processing of olfactory and mechanosensory inputs. This multimodal integration aligns with observations in other insect species and vertebrates suggesting a sophisticated neural processing strategy that could influence motor control in stick insects.

## Data Availability

The raw data supporting the conclusions of this article will be made available by the authors, without undue reservation.
