# Supplementary material for: Multi-unit recordings in the stick insect Carausius morosus reveal distinct mechanosensory and olfactory activity patterns among antennal lobe neurons
Source: Front Physiol. 2026 Jul 22;17:1873043. doi: 10.3389/fphys.2026.1873043 (PMC13437506; doi:10.3389/fphys.2026.1873043)
Supplement: Supplementary file 1 [file DataSheet1.pdf]

Supplementary Material

Multi-unit recordings in the stick insect *Carausius morosus* reveal distinct mechanosensory and olfactory activity patterns among antennal lobe neurons

Andrea Gonsek[1], Claudia Groh[2], Volker Dürr[1], Martin Strube-Bloss[1*]

[1]Department of Biological Cybernetics, Faculty of Biology, Bielefeld University, Universitaetsstr. 25, 33615 Bielefeld, Germany

[2]Behavioral Physiology and Sociobiology (Zoology II), Biocenter, University of Würzburg, Am Hubland, 97074 Würzburg, Germany

***Corresponding author: phone:** 0521 106-5532 / **e-mail: martin.strube-bloss@uni -bielefeld.de**

1. The stick insect antennal lobe consists of glomeruli-like structures forming sub compartments

We visualized the brain of adult female stick insects *Carausius morosus* (Sinéty, 1901). After dissecting the brains in cold Ringer solution, they were immediately fixed in 4% formaldehyde (Electron Microscopy Sciences #15710) for at least 18 h at 4°C. Afterwards, brains were first washed in phosphate-buffered saline (PBS, 3 × 10 min), then in PBS with 3% Triton-X 100 detergent (1 × 20 min), followed by PBS with 0.5% Triton-X 100 (2 × 10 min) all at room temperature. Brains were pre-incubated in PBS with 0.5% Triton-X 100 and 2% normal goat serum (NGS) for one hour at room temperature. To label presynaptic sites, brains were incubated with anti-synapsin antibody (1:25; CAT# 3C11 (anti SYNORF1), RIF_AB_528479, DSHB) in PBS with 0.5% Triton-X100 and 2% NGS for 6 days at 4°C. After intense rinsing in PBS (6 × 30 min), brains were incubated in 1:250 Alexa Fluor 568-conjugated goat anti-mouse secondary antibody (Cat # A-11004, Invitrogen, Thermo Fisher Scientific, Waltham, MA, USA) in PBS with 1% NGS for another 5 days at 4°C. The brains were again washed in PBS (6 × 30 min) and then dehydrated in an ascending ethanol series (30%, 50%, 70%, 90%, 95%, 2×100%, 10 min each). Finally, brains were cleared and mounted in methylsalicylate and then visualized using a laser-scanning confocal microscope (Leica TCS SP8, Leica Microsystems, Wetzlar, Germany), using a 20× objective (HC PL APO IMM CORR CS2 20×/0.75, Leica Microsystems, Wetzlar, Germany) with water-immersion and a OPSL 552 nm laser (Coherent Inc., Santa Clara, USA).

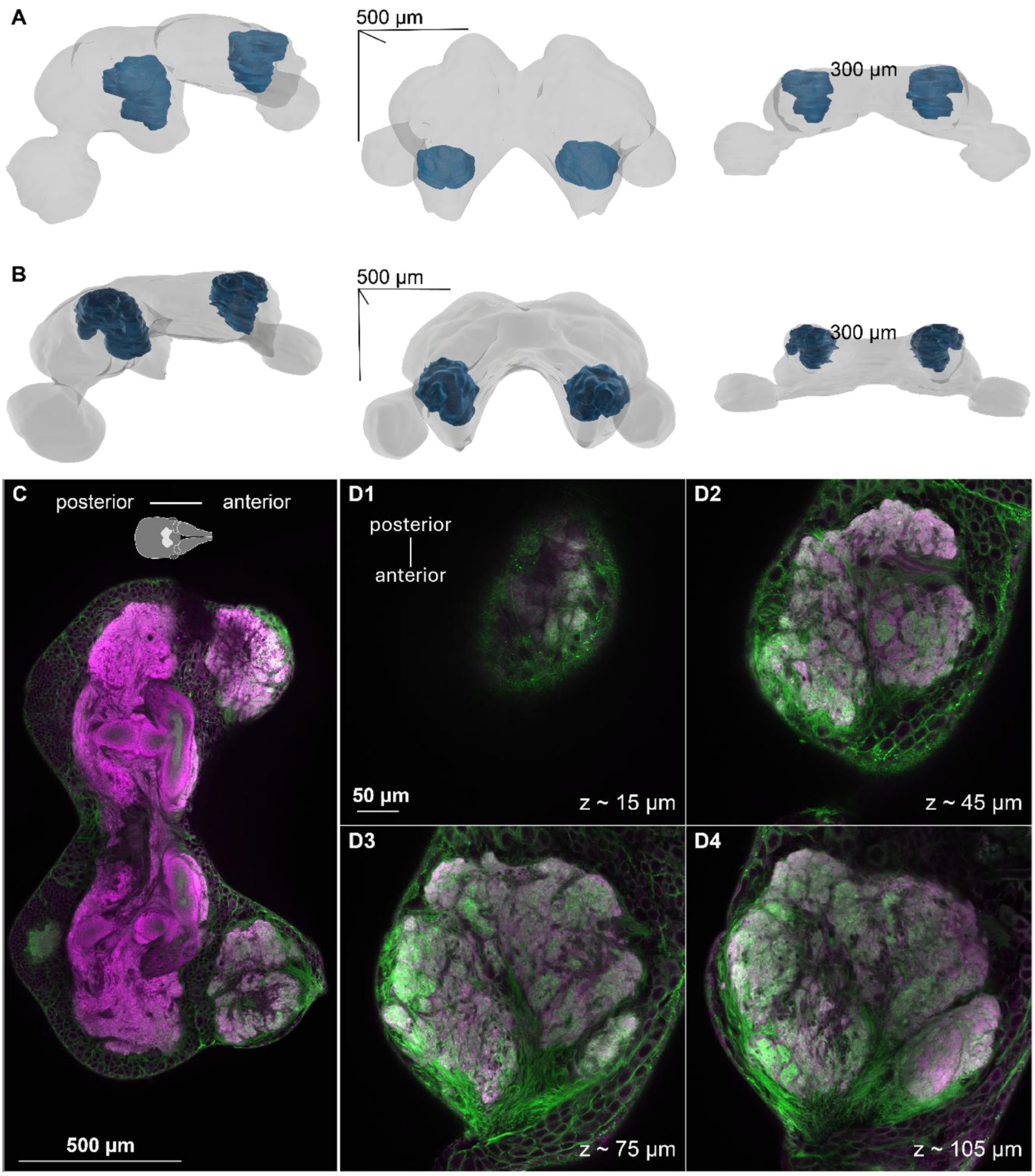

Figure S1: Antennal lobe (AL) orientation and dimension within the stick insect brain. (A) 3D reconstruction of the total brain (grey) and AL (blue) surface of synapsin wholemount stainings. Shown are the skewed view, top view, and front view (from left to right). Numbers at the verry right give the dorso-ventral elongation of the AL (300µm). (B) Same as A, but for another sample. (C) Dorsal overview of a central plane of the brain (agarose section double stained with anti-synapsin (magenta) and phalloidin (green)). The exact depth is not possible to discern due to the sectioning procedure. Inset at the top shows the brain (light gray) in relation to the head (dark gray) of the stick insect. (D1) Detailed AL scan with double stainings of anti-synapsin (magenta) and phalloidin (green) for different depths. Z-coordinate approximations were calculated from the number of the slice and the distance between slices. (D2-D4) Same as D1, but for different depths. Scale in C: 500 µm, D1 (also applies to D2-D4): 50 µm.

In addition to the synapsin whole-mount stainings, another set of brains was used to perform double-labelings against synapsin and F-actin. We dissected, fixed and washed the brains as

described above. Afterwards, brains were embedded in 5% low-melting-point agarose and sectioned horizontally at 100 µm in PBS with a vibrating microtome (Leica VT1000 S, Nussloch, Germany). Free floating agarose sections were then washed in PBS with 2% Triton-X 100 for 1 × 10 min and afterwards in PBS with 0.2% Triton-X 100 for 2 × 10 min. Sections were pre-incubated in PBS with 0.2% Triton-X 100 and 2% NGS for one hour at room temperature. Primary antibody labelling was done by incubating the sections in 1:25 mouse anti-synapsin antibodies in PBS with 0.2% Triton-X100 and 2% NGS for 2 days at 4°C. After washing in PBS (5 × 10 min), sections were incubated simultaneously for one night at 4°C in 1:250 Alexa Fluor 568-conjugated goat anti-mouse secondary antibodies with 1% NGS and in 1:200 CF633-conjugated Phalloidin to label synapsin and F-actin, respectively. Then, sections were washed in PBS (5×10 min) and transferred to 60% glycerol in PBS for one hour at room temperature. Finally, sections were mounted in 80% glycerol in PBS on slides, and scanned with confocal laser scanning microscopy, as described above.

Visualization of the brain clearly shows the antennal lobe (AL), allowing its 3D reconstruction (Fig. S1 A, B). The total depth of the AL was approximately 300 µm. A close-up zoom through the AL shows a rather inhomogeneous distribution of ordinary glomeruli (varying in shape and size), and in addition a kind of AL sub-compartments (Fig. S1 D1-4). However, our anecdotal observation was, that dorsal, glomeruli were more distinct from each other. At a larger depth, they were increasingly ambiguous. Moreover, the F-actin phalloidin staining also indicated the paths of neuronal tracts, possibly ORNs in the centre of the AL reaching into different sub-compartments and/or glomeruli. Because of that morphological variation within the AL, we may speculate that this structure is probably innervated by different receptor neurons, potentially related to different modalities

2. Recording potential AL-projection neurons

3D reconstruction of the electrode position verified that recording electrodes were positioned posterior into the AL 'neck', at a region where we expected PNs to bundle and form the AL tract (Galizia and Rössler, 2010). Four examples where the dye of the electrode (indicated in magenta) was clearly visible are shown in Figure S2. The electrode was always posterior to the AL, but its lateral position differed slightly across samples. In Figure S2A (left AL) we recorded more laterally than expected, whereas in Fig. S2B (right AL) we recorded more medially (in reference to the whole brain). Similar to Fig. S2B, Fig. S2C (left half of brain shown) also showed a more medial recording position. In Fig. S2D (right AL), the electrode was positioned medial to the right AL.

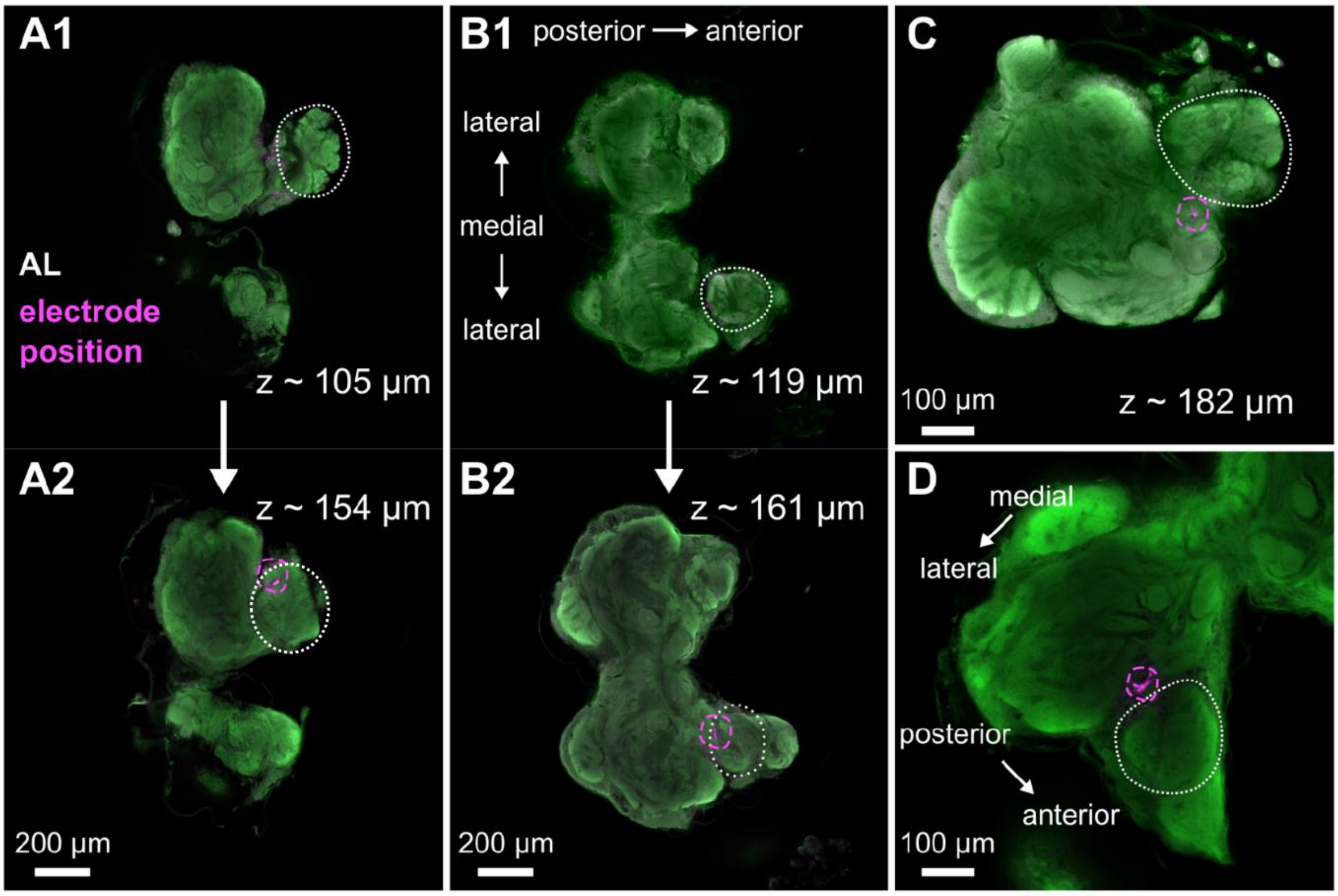

Figure S2: Visualization of the electrode position. (A)-(D) Green: autofluorescence, magenta: fluorescent dye (Alexa 647) from the electrode tip. The white dotted line indicates the position of the AL and the magenta dashed line marks the dye distribution and, thus, electrode position. The given z-coordinates of the related depths were estimated from the number of the slice and the distance between slices. Annotated axis directions in B1 and D are based on the animal axis (not the neuraxis). (A1)-(A2) Confocal scan of a post-recording sample with dye on the electrode tip. Shown are scans from two different depths (z-coordinates). A1 shows a more superficial scan, where the AL is best visible, whereas A2 shows a deeper scan where the dye was most prominent. (B1)-(B2) Same as A1-A2, but for another sample. (C) Only the left half of the brain, but same A-B. (D) Only right half of the brain shown, but otherwise same as A-B.

3. Odour valves induce valve-specific “click” sounds

The odour dependent Euclidean distances between the population vectors revealed an increased distance occurring already during the mechanosensory response window (Fig. 7). Thus, the mechanosensory activation was different for the different odours presented. Indeed, each odour stimulus and the controls were delivered through separate valves, each producing its valve-specific “click” sound, which we characterized by analysing the audio recordings of the valves (Fig. S3). Each valve shows its own characteristic power spectrum with distinct combinations of high-frequency components.

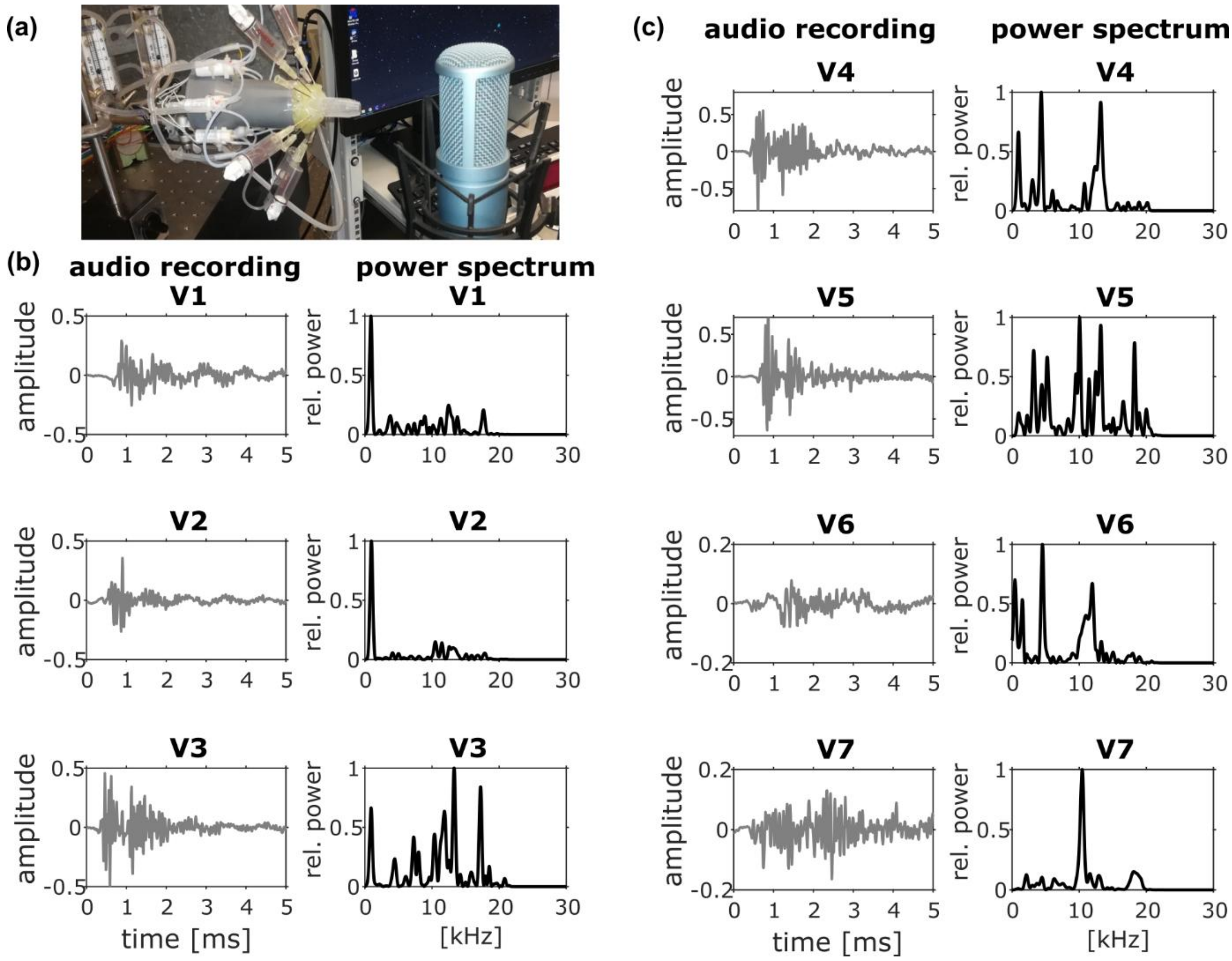

Figure S3: Characteristics of the 'valve switch'. (a) Using an ordinary condensate microphone (AKG Perception 220) we recorded the sound produced by each single valve when switched. (b) The audio recordings (left) reveal valve-specific differences among valves (V1-V3), as confirmed by their distinct power spectra (right). (c) Same as in b, but for valves 4-7 (V4-7). The peak at 1kHz, most prominent in V1 and V2 might be due to the high switching speed of the valves (about 1ms). Switching a 3-way solenoid valve of the LFAA series (LEE Hydraulische Miniaturkomponnten GmbH, Sulzbach, Germany) takes 1-15 ms, depending on the medium. Since we switch air, it should be closer to 1 ms rather than 15 ms, which would be expected for liquids.

4. AL neurones show a combinatorial coding pattern of excitation and inhibition in response to acoustical stimulation

Using an analog-to-digital converter (CED1401, Cambridge Electronic Design, Cambridge, UK) in combination with the Spike2 software (Cambridge Electronic Design, Cambridge, UK) we generated sine functions at three different frequencies (265 Hz, 600 Hz, and 900 Hz in protocol 1; 200 Hz, 450 Hz, and 750 Hz in protocol 2) and three different amplitudes (0.1V, 0.2V and 0.3V) to drive a loudspeaker. The loudspeaker was positioned 2 m away from and outside the Faraday cage to exclude electromagnetic artefacts. A silicon tube (2 cm in diameter) was attached to the speakers' front. The sound was transferred via the silicon hose into the Faraday cage, where the hose ended about 2 cm away from the animals' antennae. During the experiment, either the 9 sound stimuli of protocol 1 or those of protocol 2 were presented in a pseudorandomised sequence, 15 times each. Fig. S4 shows sound-induced responses of three example units. **Unit 1** showed a clear frequency tuning, responding most

strongly to 265 Hz. In addition, its response amplitude depended on loudness, as its response to stimulus amplitudes 0.2 V and 0.3 V was considerably stronger than to amplitude 0.1 V. **Unit 2** also showed increasingly stronger response with increasing stimulus amplitude. In addition, unit 2 showed higher sensitivity to sound frequencies 265 Hz and 900 Hz compared to the intermediate frequency of 600 Hz. The spike response of **unit 3** increased with increasing loudness, e.g. between amplitudes 0.1 V and 0.2 V of the 200 Hz. Its frequency dependence revealed a combinatorial pattern with strongest excitation to the lowest frequency (200 Hz) and inhibition in response to the highest frequency (750 Hz). We concluded that the recorded units show a complex tuning behaviour, including a frequency-dependent combinatorial pattern being excited by one frequency and inhibited by another (Fig. S4).

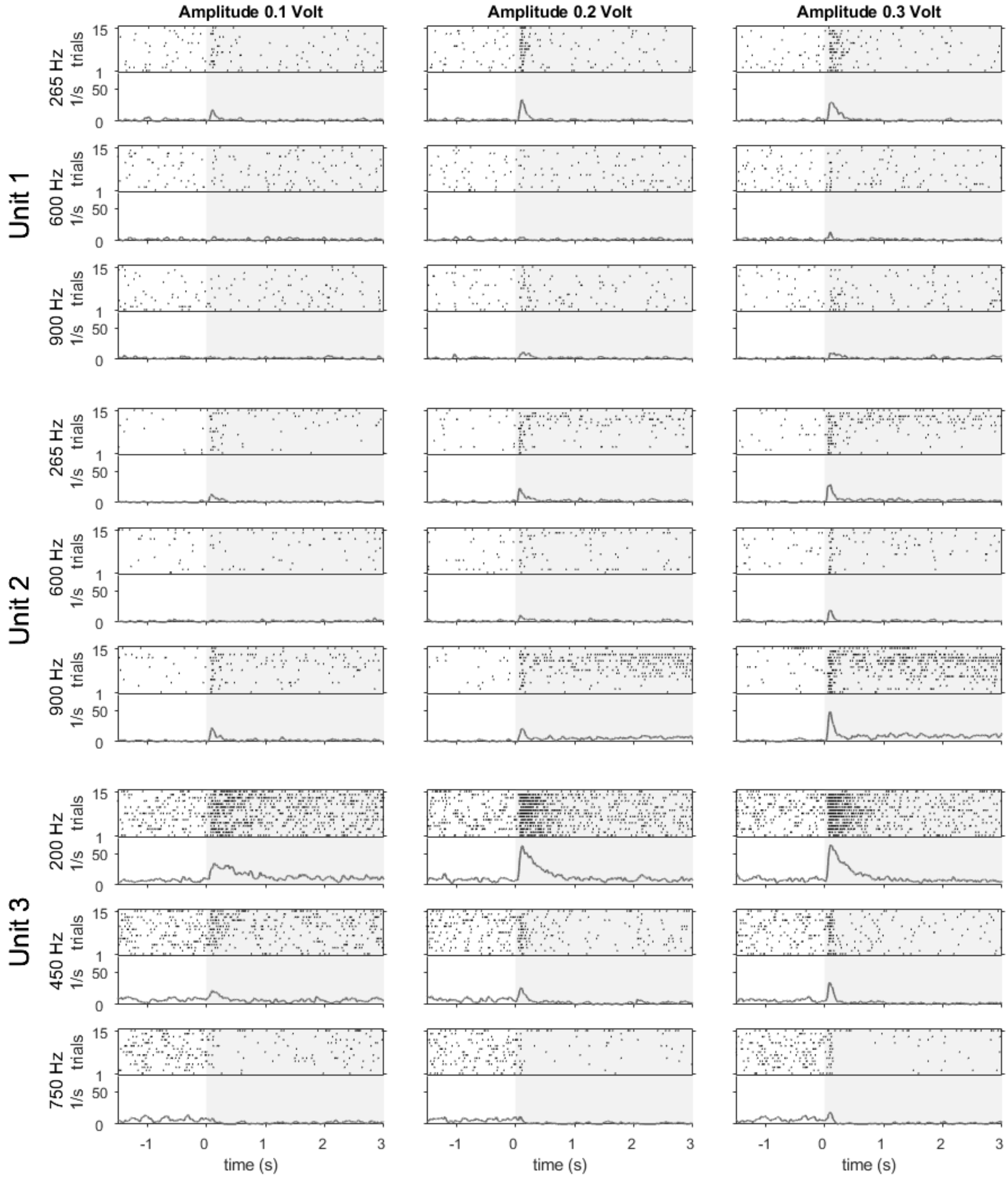

Figure S4: Sound-induced spike responses of three example units. Dot displays show 15 stimulus repetitions of nine frequency:amplitude combinations per unit. The peri-stimulus time histograms below show the averaged mean firing rate. Stimulation started at time 0 s and lasted for 3 s (light grey back ground). Frequencies were ordered in rows, intensities were ordered in columns. Example units 1 and 2 were stimulated using protocol 1, unit 3 with protocol 2.

4. EAGs show statistically different responses to the tested odour components

Table S1 ***EAG statistical analysis.*** *We used a Friedman Test followed by posthoc paired comparisons with Bonferroni correction.*

Stimulus 1	Stimulus 2	p-value
Air	Hexanal	0.0043
Air	Citral	2.7093e-17
Air	Farnesol	1
Air	Geraniol	2.5843e-10
Air	Citronellol	5.3055e-11
Hexanal	Citral	4.1141e-06
Hexanal	Farnesol	1
Hexanal	Geraniol	0.0291
Hexanal	Citronellol	0.0132
Citral	Farnesol	5.3055e-11
Citral	Geraniol	0.6188
Citral	Citronellol	1
Farnesol	Geraniol	1.3417e-05
Farnesol	Citronellol	4.1141e-06
Geraniol	Citronellol	1

5. Response onset and offset of olfactory and mechanosensory responses come from distinct distributions

Table S2 ***Response latency analysis (see Fig.5) with two-sample Kolmogorov-Smirnov tests*** *with an adjusted alpha value of 0.01 after Bonferroni (α_{corr} = 0.05/5 = 0.01 (*) and α_{corr} = 0.001/5 = 0.0002 (***)). Each distribution of olfactory response onsets was tested against the distribution of responses to the valve switch. The same was done for the offset.*

Onset		Offset	
**Stimulus**	**p-value**	**Stimulus**	**p-value**
Hexanal	1.4637e-07	Hexanal	5.9188e-06
Citral	7.5831e-10	Citral	1.2217e-06
Farnesol	1.9081e-08	Farnesol	0.0065
Geraniol	1.3404e-09	Geraniol	0.0003
Citronellol	9.0857e-10	Citronellol	0.0001

6. Odour separation during the mechanosensory and the olfactory response window

Table S3 ***Friedman test for differences in spike rate maxima of different unit populations.*** *If the Friedman test was significant, we used a Wilcoxon signed rank test with Bonferroni correction of the alpha value (α_{corr} = 0.05/10 = 0.005 (*) and α_{corr} = 0.01/10 = 0.001 (**)) for post-hoc analysis. We tested all odour pairs, p-values are shown on the right.*

Unit Population	Friedman	Condition	Wilcoxon (p)
**Odour (n=14)**			
W1	0.1712 (n.s.)		
W2	**0.0002**	hexanal vs. citral	0.9526
		hexanal vs. farnesol	0.1178
		hexanal vs. geraniol	0.8191
		hexanal vs. citronellol	0.2506
		citral vs. farnesol	0.0162
		citral vs. geraniol	0.3777
		citral vs. citronellol	0.0472
		farnesol vs. geraniol	0.6820
		farnesol vs. citronellol	0.9965
		geraniol vs. citronellol	0.8746
**Bimodal (n=82)**			
W1	**2.50739e-07**	hexanal vs. citral	0.8820
		hexanal vs. farnesol	**0.0006**
		hexanal vs. geraniol	**0.0001**
		hexanal vs. citronellol	0.0976
		citral vs. farnesol	0.0187
		citral vs. geraniol	**0.0002**
		citral vs. citronellol	0.5422
		farnesol vs. geraniol	0.7599
		farnesol vs. citronellol	0.5422
		geraniol vs. citronellol	0.0516
W2	**0.0002**	hexanal vs. citral	0.8382
		hexanal vs. farnesol	0.0083
		hexanal vs. geraniol	1.0000
		hexanal vs. citronellol	0.8136
		citral vs. farnesol	**0.0001**
		citral vs. geraniol	0.7875
		citral vs. citronellol	0.2099
		farnesol vs. geraniol	0.0116

		farnesol vs. citronellol	0.1713
		geraniol vs. citronellol	0.8610
**Bimodal+Odour(n=96)**			
W1	**1.71532e-05**	hexanal vs. citral	1.0000
		hexanal vs. farnesol	0.0288
		hexanal vs. geraniol	**0.0002**
		hexanal vs. citronellol	0.2404
		citral vs. farnesol	0.0375
		citral vs. geraniol	**0.0003**
		citral vs. citronellol	0.2844
		farnesol vs. geraniol	0.6474
		farnesol vs. citronellol	0.9089
		geraniol vs. citronellol	0.1662
W2	**2.61759e-06**	hexanal vs. citral	0.7324
		hexanal vs. farnesol	**0.0007**
		hexanal vs. geraniol	0.9872
		hexanal vs. citronellol	0.3852
		citral vs. farnesol	**0.0001**
		citral vs. geraniol	0.4127
		citral vs. citronellol	0.0218
		farnesol vs. geraniol	**0.0048**
		farnesol vs. citronellol	0.1830
		geraniol vs. citronellol	0.7048
